# Management of difficult airway in penetrating cervical spine injury

**DOI:** 10.4103/0019-5049.60501

**Published:** 2010

**Authors:** Mukesh Kumar Prasad, Ajay Kumar Sinha, Umesh Kumar Bhadani, Balbir Chabra, Kanchan Rani, Bhavana Srivastava

**Affiliations:** Department of Anaesthesiology, U.F.H.T. Medical College, Rampur Road, Haldwani, Nainital, Uttrakhand, India

**Keywords:** Difficult intubation, lateral position, malleable stylet, penetrating cervical spine injury

## Abstract

Management of airway in trauma victim with penetrating cervical/thoracic spine injury has always been a challenge to the anaesthesiologist. Stabilisation of spine during airway manipulation, to prevent any further neural damage, is of obvious concern to the anaesthesiologist. Most anaesthesiologists are not exposed to direct laryngoscopy and intubation in lateral position during their training period. Tracheal intubation in the lateral position may be unavoidable in some circumstances. Difficult airway in an uncooperative patient compounds the problem to secure airway in lateral position. We present a 46-year-old alcoholic, hypertensive, morbidly obese person who suffered a sharp instrument (screwdriver) spinal injury with anticipated difficult intubation; the case was managed successfully.

## INTRODUCTION

Penetrating trauma is one of the common admissions in casualty and the third most frequent cause of spinal injuries in adults.[1] Penetrating neck trauma poses a diagnostic and therapeutic dilemma to emergency physicians, trauma surgeons and anaesthesiologists. When penetrating trauma occurs in the neck region, it poses a special problem not only by virtue of its damaging capacity, but also due to problems in management of the airway.[2] The presence of an offending agent in the neck region compounds the problem manifold.[1,3] A thorough knowledge of the anatomy of the neck, physical assessment and current recommendations for diagnostic and therapeutic interventions are necessary for appropriate management. Expeditious decision-making is often required to prevent catastrophic airway, vascular, or neurologic sequelae.[3,4]

An unusual case of penetrating neck injury with screwdriver presented to us in the emergency department. The emergency nature of surgery with inebriated obese patient having protruding screwdriver from back of neck was the challenge to be taken by the anaesthesiologist.

## CASE REPORT

**Figure 1:**
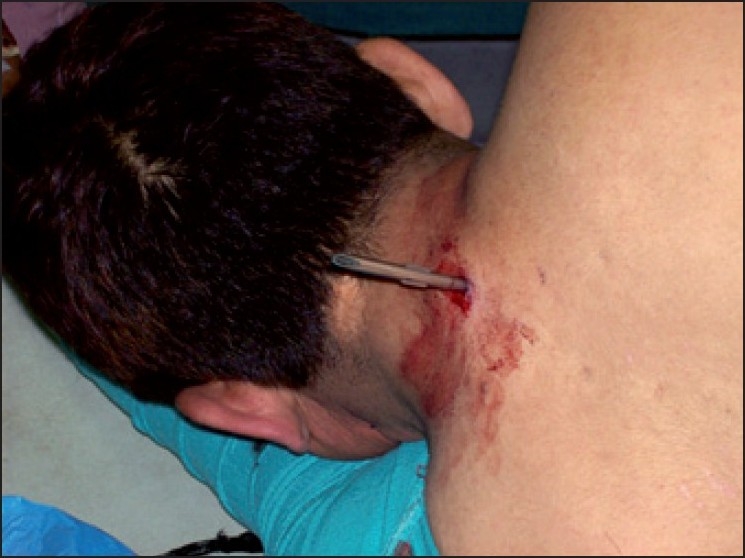
Screwdriver protruding from back of neck of patient

**Figure 2:**
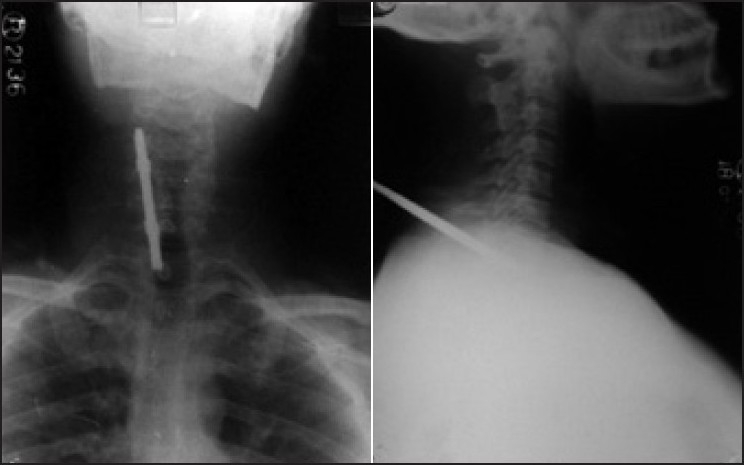
X- Ray neck - Anterior-posterior and lateral views showing screwdriver at C7-T1 interspaces

**Figure 3:**
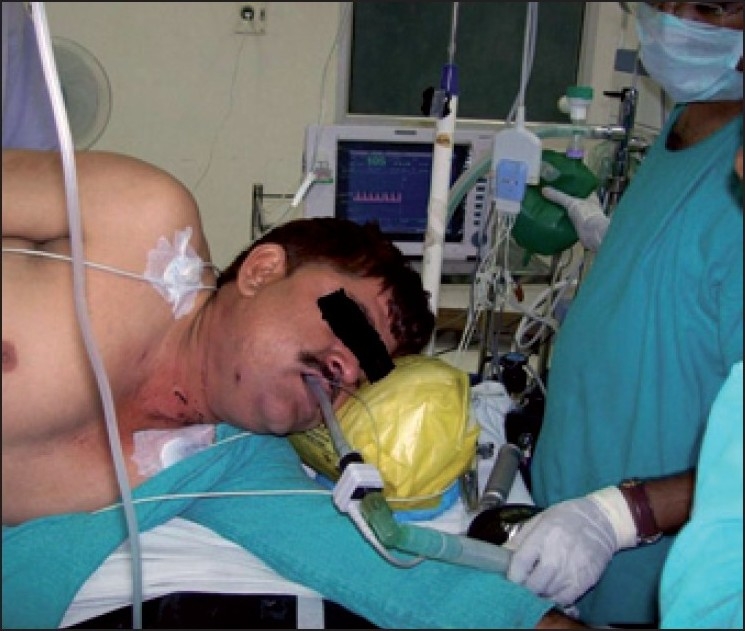
Intubation accomplished in lateral position

A 46-year-old morbidly obese man, 168 cm tall and weighing 98 kg, was brought to the emergency department with a penetrating injury in back of neck [Figure 1]. He had sustained the injury in a personal brawl with a fellow truck driver. The offending instrument (screw driver) was in between the 7^th^ cervical and 1^st^ thoracic intervertebral space *in situ*. The patient was immobilised in lateral position by cotton roles and pillow since stabilisation by cervical collar was not possible. Patient was incoherent and inebriated with smell of alcohol but answered questions satisfactorily. His pulse rate was 100/min, blood pressure 140/84, regular respiration and; he was maintaining oxygen saturation of 100%. Neurological examinations did not reveal any motor deficit at that time. There were no obvious injuries in thorax and abdomen, confirmed by radiological investigations. X-ray of neck and chest showed the point of screwdriver, which was present between 7^th^ cervical and 1^st^ thoracic intervertebral space [Figure 2]. Chest radiograph of the thorax revealed absence of pneumothorax. Lateral position and stabilisation was maintained meticulously during examination and investigations. The neurosurgeon planned to operate in prone position. The anaesthesiologist team decided to establish airway, in lateral position, in which patient was immobilised. Patient was shifted to emergency operation theatre maintaining the lateral position. His medical problems included alcoholism and hypertension on irregular treatment. Preoperative examination revealed patient was full stomach having normal intact dentition and about two-finger mouth opening. Mallampati score was III. Difficult airway situation was compounded due to patient's inebriated state, obesity, full stomach and emergency nature of surgery. Difficult airway trolley was kept ready. Establishment of airway and prevention of any neurological damage were of prime concern. Three options were considered to secure airway - fibre optic intubation, use of laryngeal mask airway and direct laryngoscopy aided with flexible stylet. Since patient was uncooperative and under influence of alcohol, ‘awake’ fibre optic intubation was not possible. Using LMA was not preferable due to full stomach situation. Direct laryngoscopy with elective use of flexible stylet was finalized. ECG, pulse oximeter, non-invasive blood pressure, temperature and provision to measure end tidal carbon dioxide were attached to patient. After preoxygenation anaesthesia was induced by propofol 2 mg/kg body weight, fentanyl 1.5 *μ*gm/kg body weight and succinylcholine 1 mg/kg body weight. After adequate anaesthetic conditions were reached, manual in line stabilisation was done. Direct laryngoscopy with (size four) Mcintosh laryngoscopic blade was performed and glottic view revealed Class 3 of Cormack and Lehane's classification. Intubation was aided with malleable 673 mm long stylet with outer diameter of 4 mm. Intubation was achieved in first attempt with size 8.0 disposable endotracheal tube [Figure 3]. Ventilation with normal peak pressure was easy.

Anaesthesia was maintained with oxygen, nitrous, halothane and vecuronium. Subsequently, patient was turned to prone position for surgical approach. Intraoperative ventilation and surgery were uneventful. Extubation and postoperative course was uneventful. Postoperative examination revealed no neurological deficit. Patient was cared in postoperative ward with uneventful recovery and discharge.

## DISCUSSION

Anaesthesiologists are not entirely familiar with airway management in the lateral position. Sudden airway compromise during surgery, when the patient is in the lateral or prone position, is hazardous because tracheal intubation can be difficult.[5] Conventional anaesthetic management for patient undergoing surgery in prone position usually starts with induction of general anaesthesia in supine position; then the patient is shifted to prone position.[6] Use of laryngeal mask airway has been used successfully in many emergency situations. Intubating laryngeal mask airway has been reported in many cases with success in clinically acceptable time. ILMA has been found to be more suitable than classical LMA in patients with recognised difficult airways.[7] In our case, patient was full stomach and a case of difficult intubation, so sudden compromise in airway during surgery in prone position was not acceptable. Awake fiberoptic intubation of trachea is considered as gold standard for tracheal instrumentation in lateral or prone position.[8,9] Fiberoptic intubation requires cooperation from patient; but patient was under the influence of alcohol, hence this option was ruled out. Moreover, awake fiberoptic intubation requires expertise and experience. Light wand assisted intubation in lateral position has also been reported but it requires experienced hands.[10] GlideScope^®^ has been used successful in ankylosing spondilitis patients with difficult airway.[11] The lateral decubitus position is associated with deterioration in laryngoscopic viewing conditions, dissociation between quality of laryngoscopic view and ease of endotracheal intubation, and an increased incidence of intubation failure has been reported.[12]

In a seminal study using manikins, Nathanson *et al*.[5] found that the lateral position was associated with longer intubation attempts and more frequent failure rates. The relatively rigid structure of the manikin limits the translatability of these data. Metal stylet has been used for management of airway in anticipated and unanticipated difficult intubation. Sandeep *et al*.[13] report use of metal stylet in emergency situations for unanticipated difficult intubations. Malleable aluminium stylet can be used as effectively as LMA or fibre optic bronchoscope in emergency situations. Management of difficult intubation in peripheral hospitals is always challenging due to lack of aids for intubation in the form of laryngeal mask airways, lighted stylets, or fibre optic bronchoscope; or the expertise to use these aids may be lacking.[14] In spite of availability of new gadgets, many a times in emergency situations we are not able to use them due to lack of expertise or lack of cooperation from patient. The malleable stylet proves handy in these situations. As far as we know, use of malleable stylet to secure difficult airway in lateral position probably has not been reported in recent literature. This comparatively inexpensive, easy-to-use aid does not require much expertise and can prove to be life-saving in emergency situations.
